# Determinants of ^18^F-NaF uptake in femoral arteries in patients with type 2 diabetes mellitus

**DOI:** 10.1007/s12350-020-02099-z

**Published:** 2020-03-17

**Authors:** Richard A. P. Takx, Ruth van Asperen, Jonas W. Bartstra, Sabine R. Zwakenberg, Jelmer M. Wolterink, Csilla Celeng, Pim A. de Jong, Joline W. Beulens

**Affiliations:** 1 Department of Radiology, University Medical Center Utrecht, Heidelberglaan 100, 3584 CX Utrecht, The Netherlands; 2 Julius Center for Health Sciences and Primary Care, University Medical Center Utrecht, Utrecht, The Netherlands; 3 Image Sciences Institute, University Medical Center Utrecht, Utrecht, The Netherlands; 4 Department of Epidemiology & Biostatistics, Amsterdam Public Health Research Institute, Vrije Universiteit, University Medical Center, Amsterdam, The Netherlands

**Keywords:** PAD, diabetes, atherosclerosis, PET, CT

## Abstract

**Background:**

The goal of this study was to investigate the potential determinants of ^18^F-NaF uptake in femoral arteries as a marker of arterial calcification in patients with type 2 diabetes and a history of arterial disease.

**Methods and Results:**

The study consisted of participants of a randomized controlled trial to investigate the effect of vitamin K2 (NCT02839044). In this prespecified analysis, subjects with type 2 diabetes and known arterial disease underwent full body ^18^F-NaF PET/CT. Target-to-background ratio (TBR) was calculated by dividing the mean SUV_max_ from both superficial femoral arteries by the SUV_mean_ in the superior vena cava (SVC) and calcium mass was measured on CT. The association between ^18^F-NaF TBR and cardiovascular risk factors was investigated using uni- and multivariate linear regression corrected for age and sex. In total, 68 patients (mean age: 69 ± 8 years; male: 52) underwent ^18^F-NaF PET/CT. Higher CT calcium mass, total cholesterol, and HbA1c were associated with higher ^18^F-NaF TBR after adjusting.

**Conclusion:**

This study shows that several modifiable cardiovascular risk factors (total cholesterol, triglycerides, HbA1c) are associated with femoral ^18^F-NaF tracer uptake in patients with type 2 diabetes.

**Electronic supplementary material:**

The online version of this article (10.1007/s12350-020-02099-z) contains supplementary material, which is available to authorized users.

## Introduction

Arterial calcification on CT is used as a marker for the presence of intimal calcification (atherosclerosis) and medial arterial calcification. Microcalcifications (< 50 µm) are observed in the early stages of the calcification process and are possibly associated with plaque rupture, whereas macrocalcifications contribute to stability of atherosclerotic plaques.^1^ In addition, microcalcifications in the internal elastic lamina and in the tunica media,^2^,^3^ may be related to arterial stiffening.^4^ CT allows to identify macrocalcifications of over 200 µm in diameter.^5^ Sodium 18F-fluoride (^18^F-NaF) PET/CT is a novel imaging technique that can visualize smaller calcifications.^6^–^10^ In the femoral arteries, ^18^F-NaF PET can visualize ongoing (medial and intimal) calcification.^11^ In the coronaries ^18^F-NaF PET is associated with high risk plaque features and plaque rupture.^6^ In patients with diabetes, additional to atherosclerosis, medial arterial calcification (MAC or Mönckeberg’s sclerosis) is highly prevalent and is associated with increased arterial stiffness, hypertension, distal symmetrical neuropathy, chronic kidney disease, cardiovascular and all-cause mortality.^12^–^14^ MAC is observed in arteries of the lower extremity across the entire length.^3^ A recent study showed that increased ^18^F-NaF PET accumulation in the femoral arteries is associated with diffuse calcium deposition and significantly correlates with cardiovascular risk factors.^11^ Several studies investigated determinants of ^18^F-NaF uptake in oncologic patients.^11^,^15^ While the use of ^18^F-NaF is increasing, the available data are limited regarding its uptake values and potential uptake modifiers in high CVD risk groups such as diabetes patients. The goal of this study is to investigate the potential determinants of ^18^F-NaF uptake as a marker of arterial calcification in patients with type 2 diabetes and a history of arterial disease.

## Method

### Study Design and Population

The study population consisted of participants of a randomized controlled trial (ClinicalTrials.gov Identifier: NCT02839044).^16^,^17^ Patients were recruited at the University Medical Center Utrecht between June 2016 and September 2018. The goal of the study was to investigate the effect of vitamin K2 supplementation on arterial calcification in patients with type 2 diabetes. Inclusion criteria were age > 40 years with type 2 diabetes and documented presence of cardiovascular disease. The presence of arterial diseases was defined as coronary artery disease, stroke, peripheral artery disease, abdominal aortic aneurysm or an ankle brachial index (ABI) of < 0.9. The study protocol was approved by the local medical ethical committee (NL53572.041.15, METC 15/571) and all patients provided written informed consent. In this prespecified analysis, baseline ^18^F-NaF PET/CT scans and patient data were used to determine the potential contributors of increased ^18^F-NaF uptake. Patient data were collected from questionnaires and available medical records. All participants completed a questionnaire on medical history, medication, smoking history, and a physical examination including height, weight and blood pressure. Non-fasting blood samples were collected to measure cholesterol levels, HbA1c levels and creatinine.

### PET/CT Imaging and Quantification

**Figure 1:**
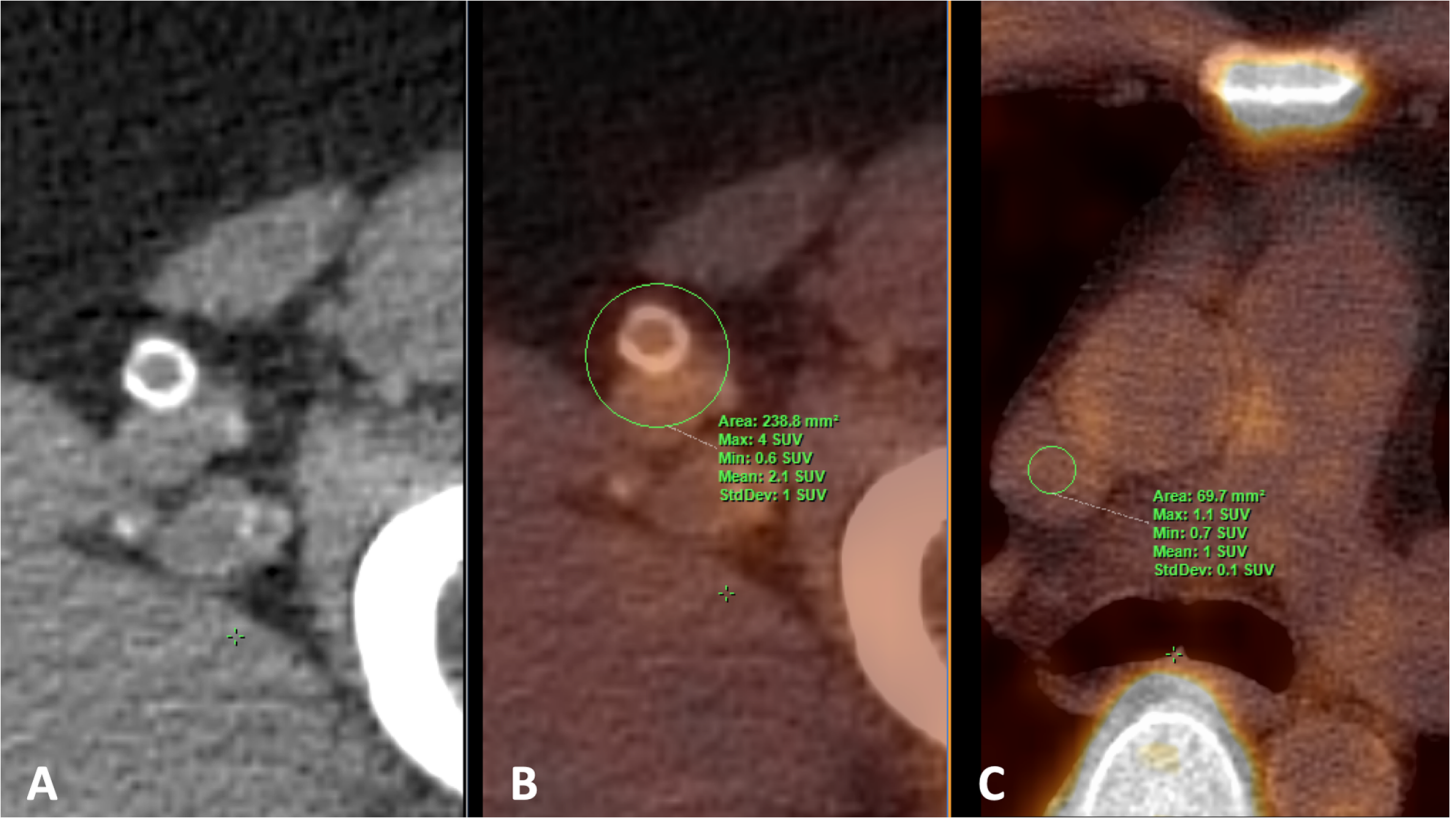
Axial CT image **A** showing circular calcification in the superficial femoral artery and fusion image **B** with a circular region of interest (ROI) drawn for quantification ^18^F-NaF SUVmax in the femoral artery. Axial fusion image (**C**) demonstrating background measurement of ^18^F-NaF SUVmean in the superior vena cava using a circular ROI

All patients underwent a full body ^18^F-NaF PET/CT scan, performed on a Siemens Biograph 40 scanner (Siemens Healthineers, Erlangen, Germany). Imaging was performed 90 min after intravenous injection of 2.0 MBq/kg ^18^F-NaF, with a maximum dosage of 200 MBq. Circular regions of interest (ROI) were drawn bilaterally around the superficial femoral arteries (see Figure 1A and B) from the bifurcation of the femoral artery to the femur condyles on every sequential axial slice to determine the ^18^F-NaF uptake. To derive the average maximum standardized uptake value (SUV_max_), maximum values measured per ROI were summed and averaged. Target-to-background ratio (TBR) was calculated by dividing the mean SUV_max_ from both femoral arteries by the SUV_mean_ from three sequential slices in the SVC (see Figure 1C), the circular ROI size was not fixed as the vena cava size differs between individuals. The femoral artery TBR was used as the measure of femoral ^18^F-NaF PET activity. To evaluate inter-rater reproducibility on radiotracer uptake ten participants were randomly selected and analyzed by thee blinded raters.

### Calcification Quantification

Non-contrast-enhanced CT scans were visually evaluated using in-house built software to identify calcium scores in the femoral arteries. A threshold of ≥ 130 Hounsfield units was used and calcification was quantified using the calcium mass equivalent score which considers calcification volume and density.

### Statistical Analyses

Normality of the data was tested using Quantile–Quantile plots. Descriptive data were expressed as mean ± standard deviation (SD) for normally distributed continuous variables, and median (Q1-Q3) for non-normally distributed continuous variables. Categorical variables are displayed as count (percentage). Reliability for TBR measurements was assessed using intraclass correlation coefficient. Univariate linear regression (unstandardized betas) was used to identify associations between all variables and ^18^F-NaF TBR. Multivariate linear analysis was performed to correct for age and sex. No adjustments were made for multiplicity of testing, and no imputation was used for missing values (only 3 patients had missing data on calcium mass). A *p* value < 0.05 was considered statistically significant. Statistical analyses were performed using SPSS software (IBM Corp, Version 24.0. Armonk, NY, USA).

## Results

### Patient Characteristics

The study population consisted of 68 participants (76% male) with a mean age 69 ± 8 years. Glucose lowering drugs were used by 60 (88%) patients, while insulin was used in 32 patients (47%). In total 25 patients used both insulin and glucose lowering drugs and only one patient was neither on insulin nor glucose lowering drugs. Fifty-two participants (77%) were treated with statins. As defined by the inclusion criteria all patients had a history of cardiovascular disease. Femoral arterial calcification on CT was present in 97% of the study population. Arterial ^18^F-NaF was measured on average on 76 slices per patient (38 per side). Baseline characteristics of the patients are reported in Table 1.

**Table 1:** Patient characteristics of the study population

Clinical characteristics	*N *= 68
Age (years)	69 ± 8
Male	52 (76)
Blood pressure	
Systolic (mmHg)	137 ± 18
Diastolic (mmHg)	72 ± 11
Body mass index (kg/m^2^)	31.2 ± 5.3
Smokers	
Former	42 (62)
Current	10 (15)
Femoral 18F-NaF PET TBR	2.2 ± 0.7
Femoral calcium mass by CT	118 (14–451)
Cholesterol	
Total (mmol/l)	4.4 [3.5–5]
LDL (mmol/l)	2.1 [1.4–2.5]
HDL(mmol/l)	1.1 ± 0.3
Triglycerides (mmol/l)	2.9 [1.6–3.4]
HbA1c (mmol/mol)	58.2 [47.3–63.0]
Serum creatine (μmol/l)	85.2 ± 23.4
History of arterial disease	
CAD	41 (60)
CVA, TIA	23 (34)
PAD	16 (24)
ABI < 0.9	18 (27)
AAA	6 (9)
Medication use	
Insulin	32 (47)
Glucose lowering drugs	60 (88)
Statins	52 (77)
Lipid lowering drugs	31 (46)
Bisphosphonates	1 (2)
Antihypertensive drugs	60 (88)
Platelet inhibitors	31 (46)
Calcium supplements	9 (13)
Vitamin D supplements	21 (31)

Values are represented as mean ± SD, median [Q1-Q3] or n (%)

*TBR*, target-to-background ratio; *LDL*, low-density lipoprotein; *HDL*, high-density lipoprotein; *HbA1c*, glycated hemoglobin; *CAD*, coronary artery disease; *CVA, TIA*, cerebrovascular disease, transient ischemic attack; *PAD*, peripheral artery disease; *ABI < 0.9*, ankle brachial index < 0.9; *AAA*, abdominal aortic aneurysm

### ^18^F-NaF PET Activity

Higher femoral calcium mass on CT, total cholesterol, and HbA1c were associated with higher ^18^F-NaF TBR (Table 2). Age, blood pressure, creatine and body mass index (BMI) were not associated to ^18^F-NaF TBR. When applying multivariate linear regression adjusting for age and sex, CT femoral calcium mass, total cholesterol and HbA1c remained significantly associated (Table 2). A trend towards significance was seen for higher triglycerides and higher ^18^F-NaF PET TBR (p = 0.052). ^18^F-NaF PET TBR was lower in patients on statins with a trend towards significance (p = 0.058). No significant differences in TBR were observed for smokers, male sex or other medication (Table 2). The intraclass correlation coefficient for inter-rater reliability of radiotracer uptake (measured as TBR) was excellent with a value of 0.98 (95% CI 0.94-0.99).

**Table 2:** Uni- and multivariate linear regression of TBR

Clinical characteristics	Univariate β (95% CI)	p value	Multivariate β (95% CI)	p value
Age (years)	0.05 (− 0.015 to 0.025)	0.614	NA	
Female sex	− 0.025 (− 0.428 to 0.349)	0.838	NA	
Blood pressure				
Systolic (mmHg)	− 0.002 (− 0.012 to 0.007)	0.644	− 0.003 (− 0.013 to 0.007)	0.516
Diastolic (mmHg)	− 0.010 (− 0.026 to 0.005)	0.189	− 0.010 (− 0.027 to 0.007)	0.229
Body mass index (kg/m^2^)	0.019 (− 0.012 to 0.050)	0.222	0.026 (− 0.008 to 0.060)	0.127
Femoral calcium mass by CT*	0.349 (0.204–0.494)	< 0.001	0.417 (0.251–0.582)	<0.001
Cholesterol				
Total (mmol/l)	0.159 (0.034–0.284)	0.013	0.168 (0.040–0.296)	0.011
LDL (mmol/l)	0.111 (− 0.071 to 0.292)	0.227	0.120 (− 0.069 to 0.308)	0.210
HDL (mmol/l)	− 0.012 (− 0.626 to 0.602)	0.969	− 0.045 (− 0.706 to 0.616)	0.891
Triglycerides (mmol/l)	0.044 (− 0.003 to 0.090)	0.064	0.048 (0.000–0.095)	0.052
HbA1c (mmol/mol)	0.011 (0.000–0.021)	0.042	0.014 (0.003–0.025)	0.015
Serum creatinine (μmol/l)	0.001 (− 0.006 to 0.008)	0.847	0.000 (− 0.008 to 0.008)	0.965
Current smokers	− 0.042 (− 0.507 to 0.424)	0.858	− 0.030 (− 0.504 to 0.444)	0.900
Medication use				
Insulin	0.245 (− 0.080 to 0.570)	0.137	0.283 (− 0.056 to 0.622)	0.100
Statins	− 0.349 (− 0.728 to 0.030)	0.070	− 0.237 (− 0.762 to 0.014)	0.058
Glucose lowering drugs	− 0.199 (− 0.708 to 0.311)	0.439	− 0.268 (− 0.812 to 0.276)	0.329
Lipid lowering drugs	0.009 (− 0.322 to 0.340)	0.957	0.014 (− 0.323 to 0.350)	0.934
Antihypertensive	− 0.146 (− 0.656 to 0.365)	0.571	− 0.136 (− 0.656 to 0.385)	0.605
Platelet inhibitors	0.155 (− 0.251 to 0.561)	0.448	0.147 (− 0.277 to 0.571)	0.490
Calcium supplements	0.383 (− 0.094 to 0.860)	0.114	0.399 (− 0.097 to 0.895)	0.113
Vitamin D supplements	0.242 (− 0.110 to 0.594)	0.175	0.234 (− 0.130 to 0.597)	0.203

Multivariate linear regression age and sex adjusted. β is per one unit change

*log (coronary calcium score plus 1)

## Discussion

This study provides several observations that contribute to our understanding of the determinants of ^18^F-NaF PET in the femoral arteries of patients with type 2 diabetes and a history of arterial disease. Firstly, we showed that femoral calcium mass on CT is positively associated with ^18^F-NaF uptake. Second, several cardiovascular risk factors including total cholesterol, triglycerides and HbA1c were associated with ^18^F-NaF uptake in patients with type 2 diabetes. Together, these data suggest that ^18^F-NaF uptake is an important imaging biomarker of arterial disease burden both related to dyslipidemia and diabetes control even in extensively treated patients.

Previous research showed that multiple cardiovascular risk factors are associated with ^18^F-NaF uptake. Derlin et al^15^ examined ^18^F-NaF in the carotid arteries in 269 oncologic patients. ^18^F-NaF uptake was significantly associated with age, male sex, hypertension and hypercholesterolemia. In a different study among 409 oncologic patients, the association between ^18^F-NaF accumulation in femoral arteries with cardiovascular risk factors and calcified plaque burden was demonstrated.^11^ In both studies, hypercholesterolemia was associated with increased ^18^F-NaF uptake, which is in line with our study. An association between HbA1c and ^18^F-NaF uptake, had been observed in healthy controls.^18^ In patients with diabetes, high HbA1c is known to be associated with osteocalcin, which supports the concept of vascular calcification in patients with diabetes.^19^ Blomberg et al^18^ investigated ^18^F-NaF uptake in the coronary arteries of 89 healthy adults. According to their results age, female sex and BMI were independent determinants of increased coronary ^18^F-NaF uptake. In our study we did not find an association between age, sex or BMI and ^18^F-NaF uptake. The differences in associations between cardiovascular determinants and ^18^F-NaF uptake compared to our study could be explained by the differences in study population (our cohort consisted predominantly out of males with a high BMI), investigated vessels beds^20^ and inclusion criteria.

^18^F-NaF uptake is significantly higher in microcalcifications compared to macrocalcifications.^21^ Irkle et al^21^ demonstrated using preclinical μPET/μCT that microcalcifications have no barriers for penetration, while ^18^F-NaF cannot penetrate into the deeper layers of macrocalcifications. Also, microcalcifications have a relatively big surface area compared to their volume. In a prior longitudinal study, we observed that areas without calcification on CT, but with increased ^18^F-NaF uptake, had more arterial calcification at follow-up.^17^ These could represent microcalcifications not yet detectable on CT.^17^ In the current study CT detected arterial calcification was related to ^18^F-NaF uptake, suggesting a significant effect of large calcium depositions on tracer uptake with clinical PET/CT systems.

An important strength of the current study is that images were acquired 90 min after tracer injection. The 90 min time-point is considered to be advantageous, because of the balance between signal-to-background ratio and patient comfort. For comparability with other studies we used TBR as our measure of ^18^F-NaF uptake for research.^15^ There is debate on how to quantify arterial calcification with NaF PET.^22^–^24^ We calculated the TBR using the average of the SUVmax of each femoral artery slice. Though SUVmax is sensitive to noise, it can also be a good measure of the maximum disease burden in a slice.^22^,^23^ By averaging (76 slices per patient) the noise effect is reduced to some extent. Others advocate the SUVmean for an individual slice,^24^ which is less limited by noise, but also averages non-diseased parts of the artery and the blood pool. Differences in PET SUV quantification will influence the observed result therefore it would be important to achieve uniform quantification methods. Other limitations are the relative small sample size and the lack of prospective outcome data.

In conclusion, we showed that cardiovascular risk factors including total cholesterol, triglycerides and HbA1c are associated with ^18^F-NaF uptake and calcification in the femoral arteries in patients with type 2 diabetes mellitus and a history of arterial disease. Our findings show that ^18^F-NaF uptake can be an imaging biomarker of arterial disease burden both related to dyslipidemia and diabetes control. Larger studies are needed to investigate whether these findings are generalizable to non-diabetic patients with cardiovascular diseases and if (further) modification of these risk factors is beneficial for ^18^F-NaF uptake and outcome.

## New Knowledge Gained

^18^F-NaF uptake may be an important arterial imaging biomarker related to dyslipidemia and diabetes control (HbA1c) even in extensively treated patients with type 2 diabetes.

## Electronic supplementary material

Below is the link to the electronic supplementary material.

Supplementary material 1 (DOCX 12 kb)

Supplementary material 2 (MP3 3557 kb)

Supplementary material 3 (PPTX 7371 kb)

## Abbreviations

^18^F-NaF: Sodium 18F-fluoride
ABI: Ankle brachial index
MAC: Medial arterial calcification
SUV: Standardized uptake value
SVC: Superior vena cava
TBR: Target-to-background ratio

## References

[1] Shioi A, Ikari Y (2018). Plaque calcification during atherosclerosis progression and regression. J Atheroscler Thromb.

[2] Narula N, Dannenberg AJ, Olin JW, Bhatt DL, Johnson KW, Nadkarni G (2018). Pathology of peripheral artery disease in patients with critical limb ischemia. J Am Coll Cardiol.

[3] Torii S, Mustapha JA, Narula J, Mori H, Saab F, Jinnouchi H (2019). Histopathologic characterization of peripheral arteries in subjects with abundant risk factors: correlating imaging with pathology. JACC Cardiovasc Imaging.

[4] Kamenskiy A, Poulson W, Sim S, Reilly A, Luo J, MacTaggart J (2018). Prevalence of calcification in human femoropopliteal arteries and its association with demographics, risk factors, and arterial stiffness. Arterioscler Thromb Vasc Biol.

[5] Sarwar A, Rieber J, Mooyaart EA, Seneviratne SK, Houser SL, Bamberg F (2008). Calcified plaque: measurement of area at thin-section flat-panel CT and 64-section multidetector CT and comparison with histopathologic findings. Radiology.

[6] Joshi NV, Vesey AT, Williams MC, Shah AS, Calvert PA, Craighead FH (2014). 18F-fluoride positron emission tomography for identification of ruptured and high-risk coronary atherosclerotic plaques: A prospective clinical trial. Lancet.

[7] (7) Al-Zaghal A, Mehdizadeh Seraj S, Werner TJ, Gerke O, Hoilund-Carlsen PF, Alavi A. Assessment of physiological intracranial calcification in healthy adults using (18)F-NaF PET/CT. J Nucl Med 2018. 10.2967/jnumed.118.213678 30002111

[8] Krishnan S, Otaki Y, Doris M, Slipczuk L, Arnson Y, Rubeaux M (2017). Molecular imaging of vulnerable coronary plaque: A pathophysiologic perspective. J Nucl Med.

[9] Hop H, de Boer SA, Reijrink M, Kamphuisen PW, de Borst MH, Pol RA (2019). (18)F-sodium fluoride positron emission tomography assessed microcalcifications in culprit and non-culprit human carotid plaques. J Nucl Cardiol.

[10] Bellinge JW, Francis RJ, Majeed K, Watts GF, Schultz CJ (2018). In search of the vulnerable patient or the vulnerable plaque: (18)F-sodium fluoride positron emission tomography for cardiovascular risk stratification. J Nucl Cardiol.

[11] Janssen T, Bannas P, Herrmann J, Veldhoen S, Busch JD, Treszl A (2013). Association of linear (1)(8)F-sodium fluoride accumulation in femoral arteries as a measure of diffuse calcification with cardiovascular risk factors: A PET/CT study. J Nucl Cardiol.

[12] Ho CY, Shanahan CM (2016). Medial arterial calcification: An overlooked player in peripheral arterial disease. Arterioscler Thromb Vasc Biol.

[13] Lanzer P, Boehm M, Sorribas V, Thiriet M, Janzen J, Zeller T (2014). Medial vascular calcification revisited: Review and perspectives. Eur Heart J.

[14] Jeffcoate WJ, Rasmussen LM, Hofbauer LC, Game FL (2009). Medial arterial calcification in diabetes and its relationship to neuropathy. Diabetologia.

[15] Derlin T, Wisotzki C, Richter U, Apostolova I, Bannas P, Weber C (2011). In vivo imaging of mineral deposition in carotid plaque using 18F-sodium fluoride PET/CT: Correlation with atherogenic risk factors. J Nucl Med.

[16] Zwakenberg SR, de Jong PA, Bartstra JW, van Asperen R, Westerink J, de Valk H (2019). The effect of menaquinone-7 supplementation on vascular calcification in patients with diabetes: A randomized, double-blind, placebo-controlled trial. Am J Clin Nutr.

[17] (17) den Harder AM, Wolterink JM, Bartstra JW, Spiering W, Zwakenberg SR, Beulens JW et al. Vascular uptake on (18)F-sodium fluoride positron emission tomography: precursor of vascular calcification? J Nucl Cardiol 2020. 10.1007/s12350-020-02031-5 PMC8648691 31975332

[18] Blomberg BA, Thomassen A, de Jong PA, Lam MGE, Diederichsen ACP, Olsen MH (2017). Coronary fluorine-18-sodium fluoride uptake is increased in healthy adults with an unfavorable cardiovascular risk profile: Results from the CAMONA study. Nucl Med Commun.

[19] Flammer AJ, Gossl M, Li J, Matsuo Y, Reriani M, Loeffler D (2012). Patients with an HbA1c in the prediabetic and diabetic range have higher numbers of circulating cells with osteogenic and endothelial progenitor cell markers. J Clin Endocrinol Metab.

[20] O’Neill WC, Han KH, Schneider TM, Hennigar RA (2015). Prevalence of nonatheromatous lesions in peripheral arterial disease. Arterioscler Thromb Vasc Biol.

[21] Irkle A, Vesey AT, Lewis DY, Skepper JN, Bird JL, Dweck MR (2015). Identifying active vascular microcalcification by (18)F-sodium fluoride positron emission tomography. Nat Commun.

[22] Blomberg BA, Thomassen A, de Jong PA, Simonsen JA, Lam MG, Nielsen AL (2015). Impact of personal characteristics and technical factors on quantification of sodium 18F-fluoride uptake in human arteries: Prospective evaluation of healthy subjects. J Nucl Med.

[23] Blomberg BA, Thomassen A, Takx RA, Vilstrup MH, Hess S, Nielsen AL (2014). Delayed sodium 18F-fluoride PET/CT imaging does not improve quantification of vascular calcification metabolism: Results from the CAMONA study. J Nucl Cardiol.

[24] (24) Sorci O, Batzdorf AS, Mayer M, Rhodes S, Peng M, Jankelovits AR et al. (18)F-sodium fluoride PET/CT provides prognostic clarity compared to calcium and Framingham risk scoring when addressing whole-heart arterial calcification. Eur J Nucl Med Mol Imaging 2019. 10.1007/s00259-019-04590-3 31734781

