# Supplementary material for: Determinants of 18F-NaF uptake in femoral arteries in patients with type 2 diabetes mellitus
Source: J Nucl Cardiol. 2020 Mar 17;28(6):2700–5. doi: 10.1007/s12350-020-02099-z (PMC8709815; doi:10.1007/s12350-020-02099-z)
Supplement: Supplementary file 1 — Supplementary material 1 (DOCX 12 kb) [file 12350_2020_2099_MOESM1_ESM.docx]

Twitter summary

^This study suggests that 18F-NaF uptake may be an important arterial imaging biomarker related to dyslipidemia and HbA1c even in extensively treated patients with type 2 diabetes.^
