# Supplementary material for: Determinants of 18F-NaF uptake in femoral arteries in patients with type 2 diabetes mellitus
Source: J Nucl Cardiol. 2020 Mar 17;28(6):2700–5. doi: 10.1007/s12350-020-02099-z (PMC8709815; doi:10.1007/s12350-020-02099-z)
Supplement: Supplementary file 3 — Supplementary material 3 (PPTX 7371 kb) [file 12350_2020_2099_MOESM3_ESM.pptx]

## Slide 1
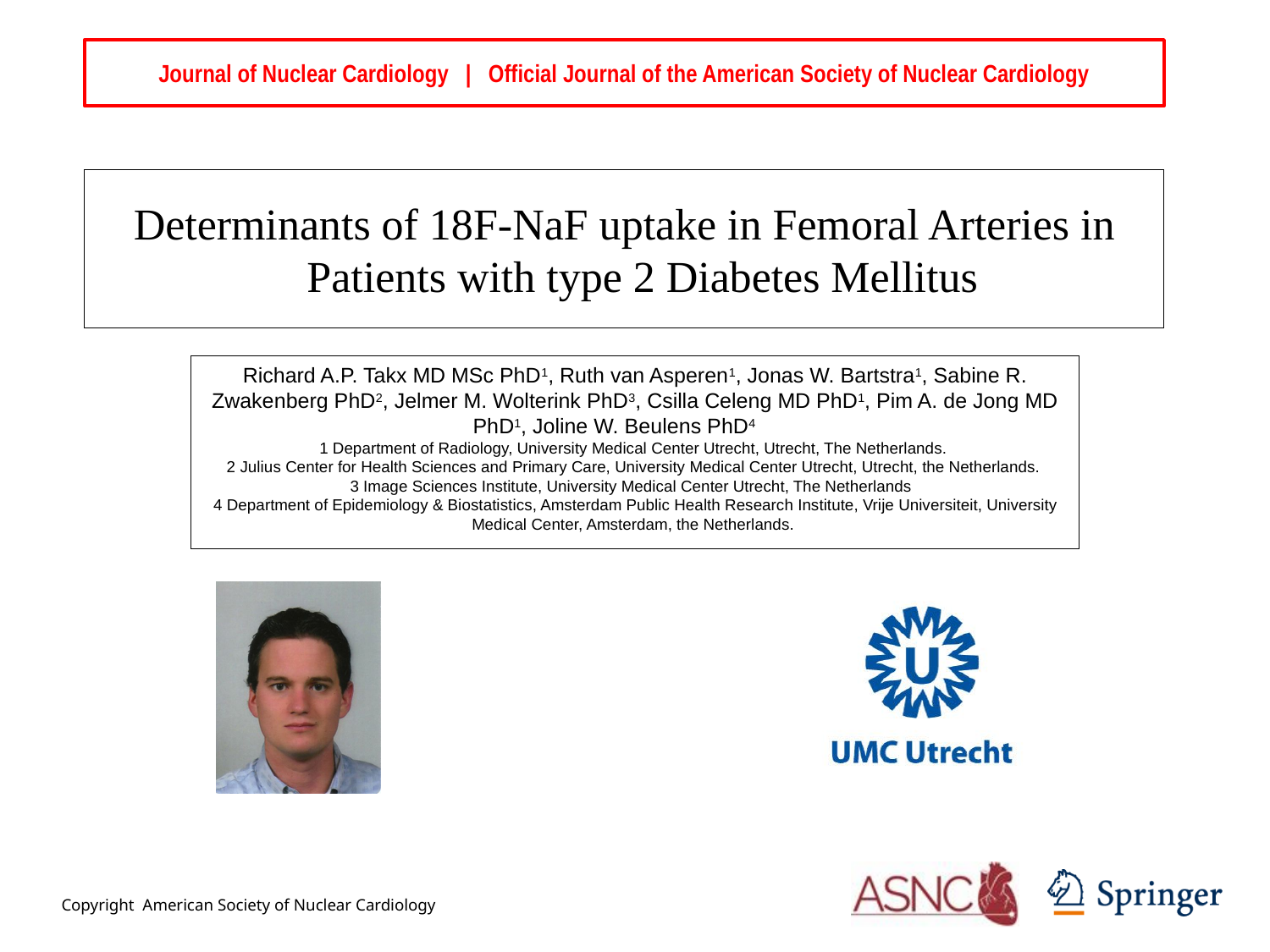

Journal of Nuclear Cardiology | Official Journal of the American Society of Nuclear Cardiology
# Determinants of 18F-NaF uptake in Femoral Arteries in Patients with type 2 Diabetes Mellitus
Richard A.P. Takx MD MSc PhD1, Ruth van Asperen1, Jonas W. Bartstra1, Sabine R. Zwakenberg PhD2, Jelmer M. Wolterink PhD3, Csilla Celeng MD PhD1, Pim A. de Jong MD PhD1, Joline W. Beulens PhD4
1 Department of Radiology, University Medical Center Utrecht, Utrecht, The Netherlands.
 2 Julius Center for Health Sciences and Primary Care, University Medical Center Utrecht, Utrecht, the Netherlands.
3 Image Sciences Institute, University Medical Center Utrecht, The Netherlands
4 Department of Epidemiology & Biostatistics, Amsterdam Public Health Research Institute, Vrije Universiteit, University Medical Center, Amsterdam, the Netherlands.
Copyright American Society of Nuclear Cardiology

## Slide 2
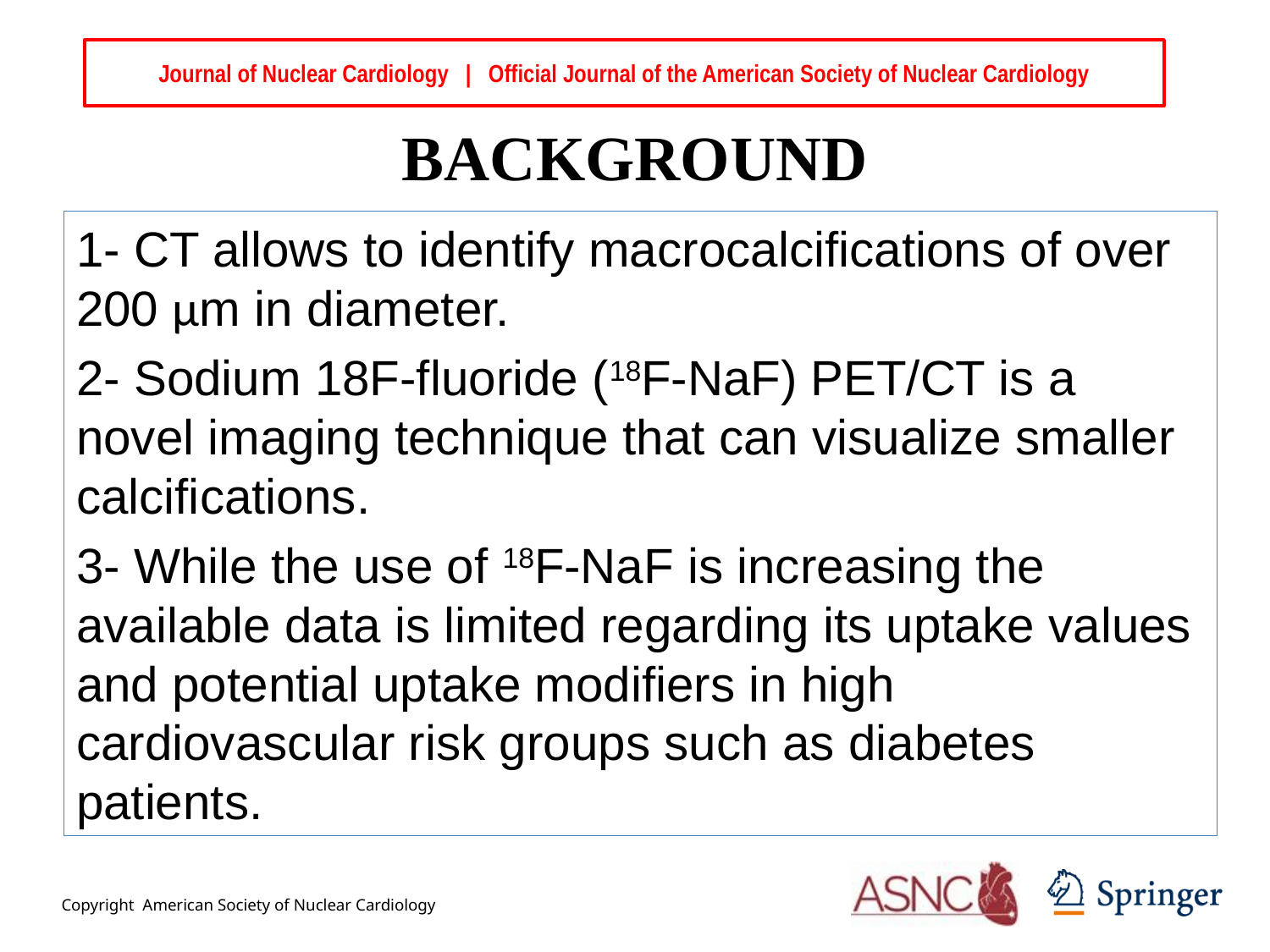

Journal of Nuclear Cardiology | Official Journal of the American Society of Nuclear Cardiology
# BACKGROUND
1- CT allows to identify macrocalcifications of over 200 µm in diameter.
2- Sodium 18F-fluoride (18F-NaF) PET/CT is a novel imaging technique that can visualize smaller calcifications.
3- While the use of 18F-NaF is increasing the available data is limited regarding its uptake values and potential uptake modifiers in high cardiovascular risk groups such as diabetes patients.
Copyright American Society of Nuclear Cardiology

## Slide 3
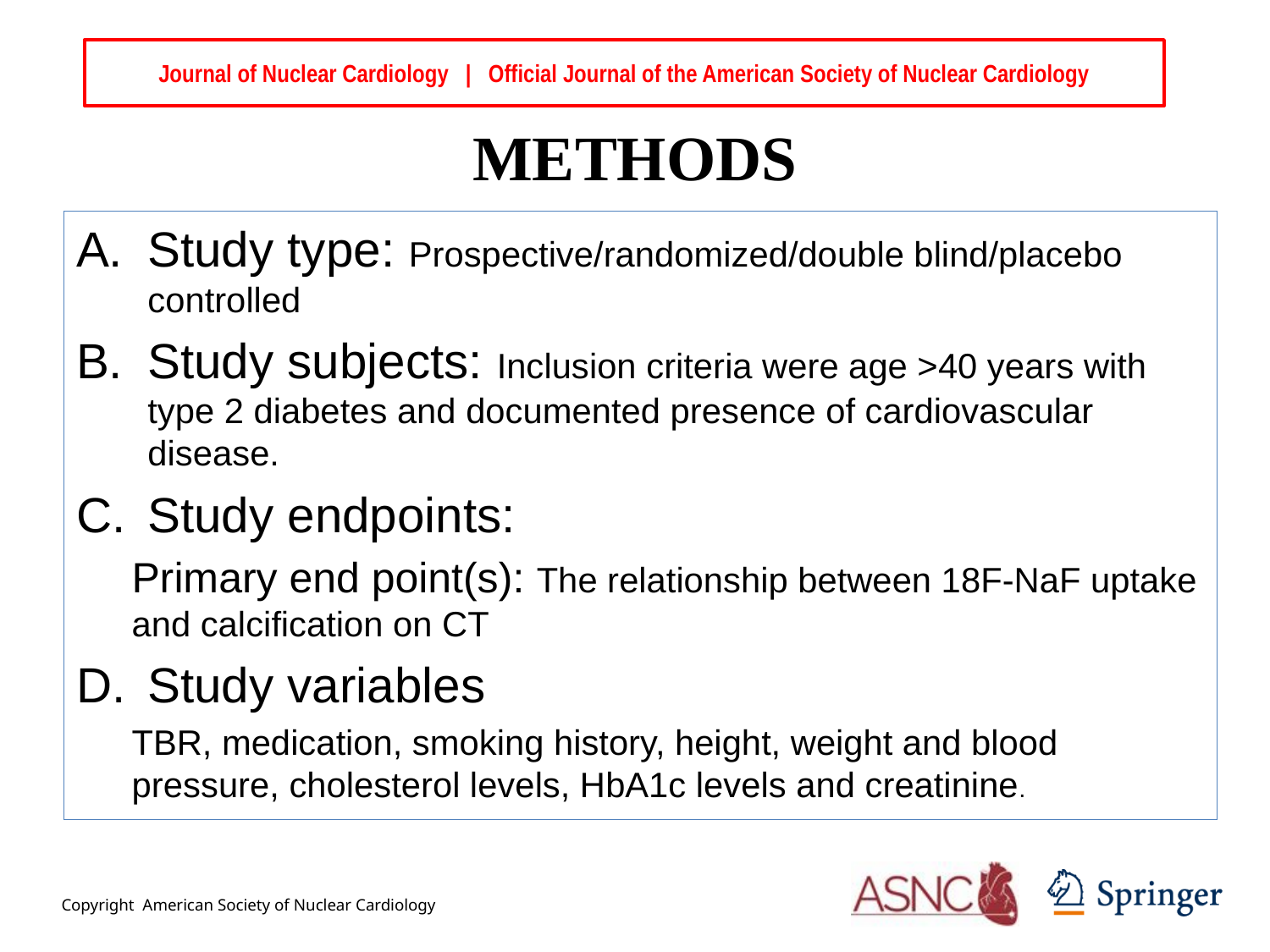

Journal of Nuclear Cardiology | Official Journal of the American Society of Nuclear Cardiology
# METHODS
Study type: Prospective/randomized/double blind/placebo controlled
Study subjects: Inclusion criteria were age >40 years with type 2 diabetes and documented presence of cardiovascular disease.
Study endpoints:
Primary end point(s): The relationship between 18F-NaF uptake and calcification on CT
Study variables
TBR, medication, smoking history, height, weight and blood pressure, cholesterol levels, HbA1c levels and creatinine.
Copyright American Society of Nuclear Cardiology

## Slide 4
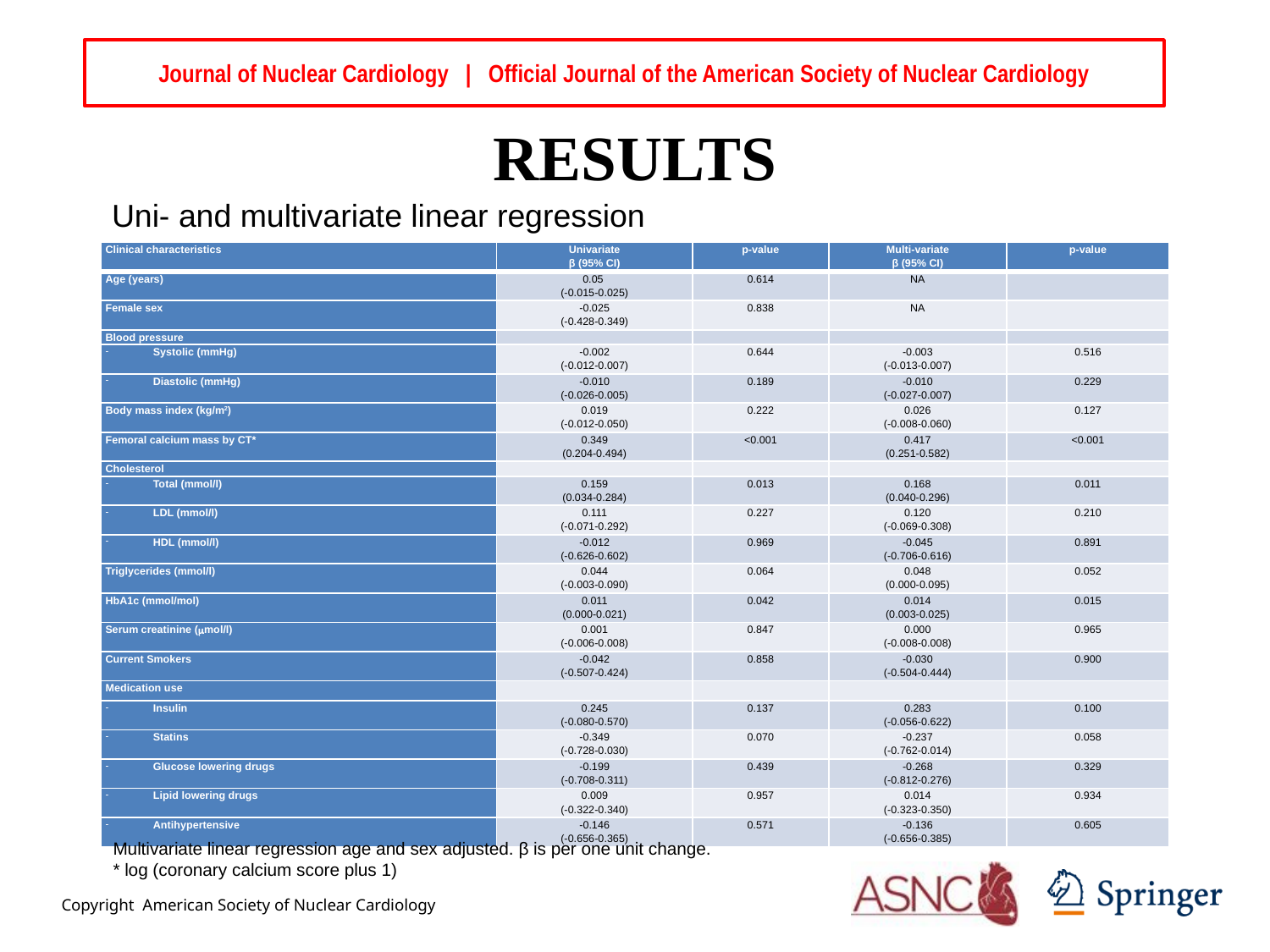

Journal of Nuclear Cardiology | Official Journal of the American Society of Nuclear Cardiology
# RESULTS
Uni- and multivariate linear regression
| Clinical characteristics | Univariate β (95% CI) | p-value | Multi-variate β (95% CI) | p-value |
| --- | --- | --- | --- | --- |
| Age (years) | 0.05 (-0.015-0.025) | 0.614 | NA | |
| Female sex | -0.025 (-0.428-0.349) | 0.838 | NA | |
| Blood pressure | | | | |
| Systolic (mmHg) | -0.002 (-0.012-0.007) | 0.644 | -0.003 (-0.013-0.007) | 0.516 |
| Diastolic (mmHg) | -0.010 (-0.026-0.005) | 0.189 | -0.010 (-0.027-0.007) | 0.229 |
| Body mass index (kg/m2) | 0.019 (-0.012-0.050) | 0.222 | 0.026 (-0.008-0.060) | 0.127 |
| Femoral calcium mass by CT\* | 0.349 (0.204-0.494) | <0.001 | 0.417 (0.251-0.582) | <0.001 |
| Cholesterol | | | | |
| Total (mmol/l) | 0.159 (0.034-0.284) | 0.013 | 0.168 (0.040-0.296) | 0.011 |
| LDL (mmol/l) | 0.111 (-0.071-0.292) | 0.227 | 0.120 (-0.069-0.308) | 0.210 |
| HDL (mmol/l) | -0.012 (-0.626-0.602) | 0.969 | -0.045 (-0.706-0.616) | 0.891 |
| Triglycerides (mmol/l) | 0.044 (-0.003-0.090) | 0.064 | 0.048 (0.000-0.095) | 0.052 |
| HbA1c (mmol/mol) | 0.011 (0.000-0.021) | 0.042 | 0.014 (0.003-0.025) | 0.015 |
| Serum creatinine (μmol/l) | 0.001 (-0.006-0.008) | 0.847 | 0.000 (-0.008-0.008) | 0.965 |
| Current Smokers | -0.042 (-0.507-0.424) | 0.858 | -0.030 (-0.504-0.444) | 0.900 |
| Medication use | | | | |
| Insulin | 0.245 (-0.080-0.570) | 0.137 | 0.283 (-0.056-0.622) | 0.100 |
| Statins | -0.349 (-0.728-0.030) | 0.070 | -0.237 (-0.762-0.014) | 0.058 |
| Glucose lowering drugs | -0.199 (-0.708-0.311) | 0.439 | -0.268 (-0.812-0.276) | 0.329 |
| Lipid lowering drugs | 0.009 (-0.322-0.340) | 0.957 | 0.014 (-0.323-0.350) | 0.934 |
| Antihypertensive | -0.146 (-0.656-0.365) | 0.571 | -0.136 (-0.656-0.385) | 0.605 |
Multivariate linear regression age and sex adjusted. β is per one unit change.
* log (coronary calcium score plus 1)
Copyright American Society of Nuclear Cardiology

## Slide 5
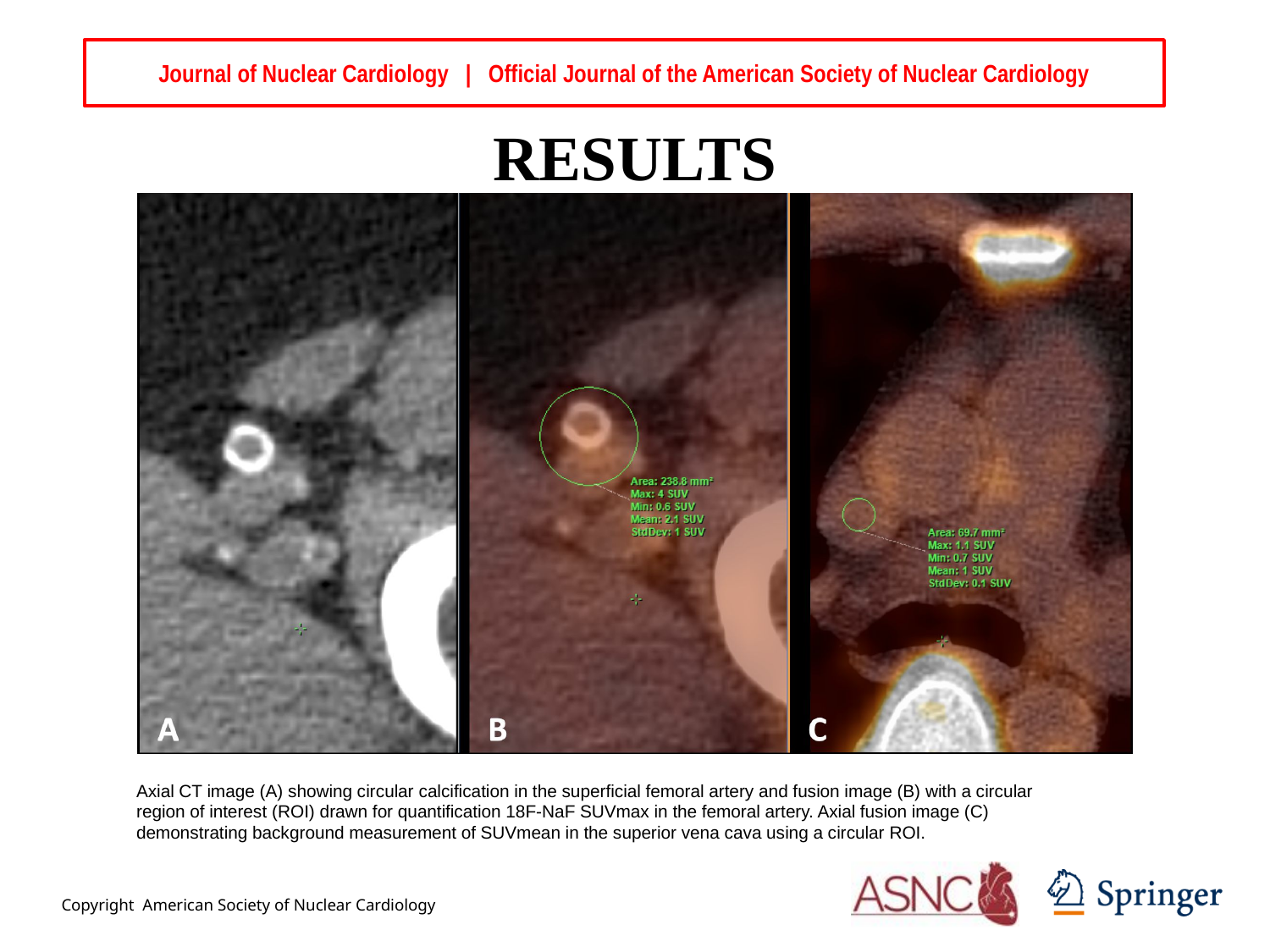

Journal of Nuclear Cardiology | Official Journal of the American Society of Nuclear Cardiology
# RESULTS
Axial CT image (A) showing circular calcification in the superficial femoral artery and fusion image (B) with a circular region of interest (ROI) drawn for quantification 18F-NaF SUVmax in the femoral artery. Axial fusion image (C) demonstrating background measurement of SUVmean in the superior vena cava using a circular ROI.
Copyright American Society of Nuclear Cardiology

## Slide 6
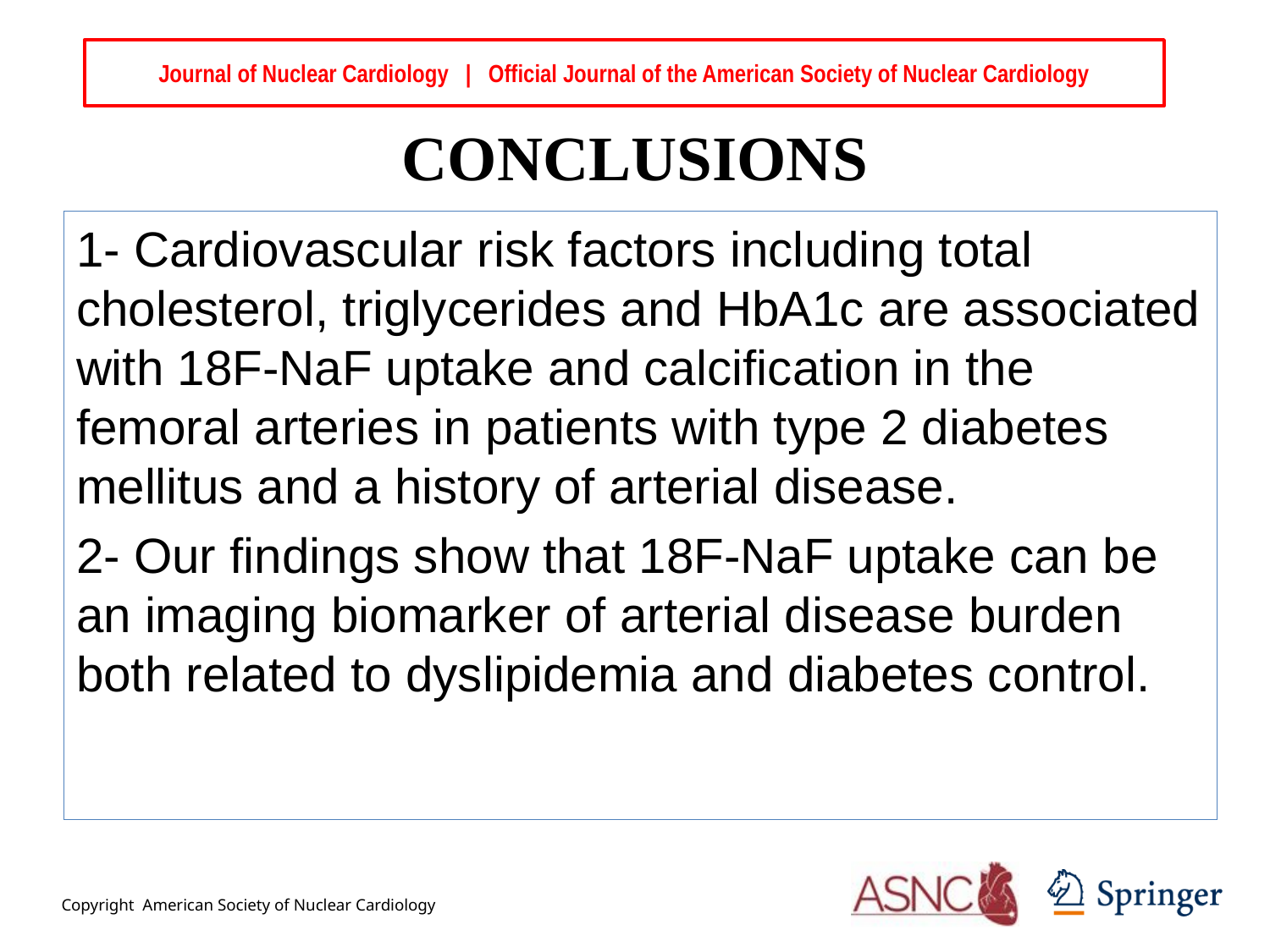

Journal of Nuclear Cardiology | Official Journal of the American Society of Nuclear Cardiology
# CONCLUSIONS
1- Cardiovascular risk factors including total cholesterol, triglycerides and HbA1c are associated with 18F-NaF uptake and calcification in the femoral arteries in patients with type 2 diabetes mellitus and a history of arterial disease.
2- Our findings show that 18F-NaF uptake can be an imaging biomarker of arterial disease burden both related to dyslipidemia and diabetes control.
Copyright American Society of Nuclear Cardiology
